# Medical students are afraid to include abortion in their future practices: in-depth interviews in Maharastra, India

**DOI:** 10.1186/s12909-016-0532-5

**Published:** 2016-01-12

**Authors:** Susanne Sjöström, Birgitta Essén, Kristina Gemzell-Danielsson, Marie Klingberg-Allvin

**Affiliations:** Division of Obstetrics and Gynecology, Department of Women’s and Children’s Health, Karolinska Institutet, 171 76 Stockholm, Sweden; Department of Women’s and Children’s Health/, International Maternal and Child Health, Uppsala University, 751 85 Uppsala, Sweden; School of Health and Social Sciences, Dalarna University, 791 88 Falun, Sweden

**Keywords:** Medical abortion, Mid-level provision, Medical education, Legal issues

## Abstract

**Background:**

Unsafe abortions are estimated to cause eight per-cent of maternal mortality in India. Lack of providers, especially in rural areas, is one reason unsafe abortions take place despite decades of legal abortion. Education and training in reproductive health services has been shown to influence attitudes and increase chances that medical students will provide abortion care services in their future practice. To further explore previous findings about poor attitudes toward abortion among medical students in Maharastra, India, we conducted in-depth interviews with medical students in their final year of education.

**Method:**

We used a qualitative design conducting in-depth interviews with twenty-three medical students in Maharastra applying a topic guide. Data was organized using thematic analysis with an inductive approach.

**Results:**

The participants described a fear to provide abortion in their future practice. They lacked understanding of the law and confused the legal regulation of abortion with the law governing gender biased sex selection, and concluded that abortion is illegal in Maharastra. The interviewed medical students’ attitudes were supported by their experiences and perceptions from the clinical setting as well as traditions and norms in society. Medical abortion using mifepristone and misoprostol was believed to be unsafe and prohibited in Maharastra. The students perceived that nurse-midwives were knowledgeable in Sexual and Reproductive Health and many found that they could be trained to perform abortions in the future.

**Conclusions:**

To increase chances that medical students in Maharastra will perform abortion care services in their future practice, it is important to strengthen their confidence and knowledge through improved medical education including value clarification and clinical training.

## Background

Maternal mortality, i.e. death of a woman whilst pregnant or within 42 days of delivery or termination of pregnancy, was estimated to cause 293,000 deaths globally in 2013. Unsafe abortion is accredited around 43,000 (15 %) of those deaths [1]. Despite a substantial reduction in the maternal mortality ratio (MMR) in India during the past decades, especially in urban areas, WHO estimates that 50,000 maternal deaths occurred in the country in 2013 [2]. Abortion has been legal in India since 1971, regulated in the medical termination of pregnancy (MTP) act of 1971 [3], yet abortion-related deaths are believed to be common. Government statistics from 2011 report that 620,472 abortions took place in India in 2010–2011, and in 2012, 8,4 % of medically certified deaths were attributed to abortion [4, 5]. It has been argued that these numbers are underestimates as only legal abortions are reported. The Consortium on National Consensus for Medical Abortion in India estimated that around 11 million abortions occured yearly and that 20,000 deaths are related to unsafe abortion [6, 7]. Three quarters of maternal deaths are reported to occur in rural areas, especially of poorer states, and it was more likely to seek consultation in the community than at health facilities in those cases [6]. Shortage of trained health care providers and lack of properly equipped health facilities are common barriers to safe abortion, especially in rural areas [8–10]. The WHO recommends that abortion is provided at the lowest level of the health care system, and task-shifting of abortion care to non-physician providers is suggested as a way to increase womens’ access to safe abortion [11]. Midlevel provision of medical and aspiration abortion has been shown to be feasible in India, and amendments to the law to approve midlevel provision of abortion care services are currently under consideration by the Ministry of Health and Family Welfare [12, 13]. Medical abortion using mifepristone and misoprostol is approved in India up to 63 days of gestation, and has been shown to be safe, reliable and effective [14], but awareness and knowledge is generally low among women and physicians, and implementation has been slow, especially in the public sector [15, 16].

Approximately 90 % of all global abortion-related mortality and morbidity could be averted by the use of effective contraception [17]. In India, knowledge of contraception is reported to be nearly universal and the most commonly adopted method is female sterilization [18]. It has been estimated that around 57 % of married women use any contraception and the unmet need for contraception, i.e. the percentage of women who want to stop or delay childbearing but who are not using any method of contraception, was 14 % in 2010. Should sexually active unmarried women have been included in the study, the unmet need was expected to be higher [19]. A recently published study from Northern India found large discrepancies between the knowledge about contraception between literate and illiterate women. Lack of knowledge and fear of side effects, as well as religious belief and partner opposition were the most common reasons reported for not using contraception. In this study over 90 % of respondents conveyed their desire to use a family-planning method after counselling [20]. Under-use of family-planning methods may also be influenced by limited knowledge and poor attitudes regarding contraceptive methods among providers [18, 21, 22].

Son preference is persistent in the Indian society and sex ratios are decreasing in many Indian states according to the 2011 census [23]. The Pre-Conception and Pre-Natal Diagnostic Technique Act of 2003 (PC-PNDT) that regulate the use of radiology, in-vitro fertilization and genetic laboratories to determine the sex of the fetus, is often interlinked with the MTP act regulating abortion by authorities and providers [24]. Actions taken to implement the law regulating sex-selective abortion have created confusion among physicians regarding provision of abortion on legal grounds, and many avoid providing abortions, especially in the second trimester [25, 26]. To address the challenge of limiting gender biased sex selection while protecting women’s access to safe abortion the Government of India has recently published a guidance handbook [27].

Medical education is regulated by the Medical Council of India and consists of 4.5 years mainly theoretical education, followed by a one-year practical rotating internship including two months of obstetrics and gynecology, leading to the degree Bachelor of Medicine and Bachelor of Surgery (MBBS). Knowledge and clinical training in family planning including contraception and abortion is stressed in the national curricula for medical education [28].

Attitudes toward contraception and abortion are influenced by education and socio-demographic factors, and significantly correlate with medical students’ willingness to provide safe family planning services in the future [29, 30, 31]. Training in comprehensive abortion care influences attitudes toward abortion and increases the likelihood that gynaecologists will perform abortions in their practice We recently presented a survey of Maharastra’s medical students showing that negative attitudes regarding women’s rights to have an abortion on demand, as well as misconceptions about legal regulation governing termination of pregnancy were common, and that clinical training in abortion care services was rare [32]. The present study complements the findings of our previous survey and aims to explore the attitudes and perceptions toward abortion care services, medical abortion and task shifting in abortion care among medical students in their internship year, in Maharastra, India.

## Method

### Study design

We used a qualitative design conducting in-depth interviews with twenty-three medical students in Maharastra applying a topic guide. Data was organized using thematic analysis with an inductive approach.

### Setting

Maharashtra is India’s second most populous state with 112 million inhabitants, and the third largest by area. The state capital Mumbai is the largest city in India with around 13.3 million inhabitants, yet a slight majority of the state’s inhabitants live in rural areas. In 2010–2012 the maternal mortality rate in Maharastra of 178 was around half of the national average, and according to data from 2001–2003 the maternal mortality due to abortion was 3 % for a group of seven well-off states including Maharastra [33]. Child sex ratio (0–6 years) was 883 females per 1000 males (national average 914) in the 2011 census, and the gender gap increased in the decade 2001–2011 [5, 34]. There are 44 medical colleges offering the MBBS degree to 5945 students in Maharastra of which 19 are run by the government [34].

### Study participants

In-depth interviews were conducted with 23 medical students in their last year of training leading to the MBBS degree (internship). The students attended 6 different colleges (4 government (*n* = 15) and 2 private (*n* = 8)) in urban and peri-urban areas. The schools were purposively sampled among 28 private and public colleges that had previously engaged in a survey on attitudes to abortion. Purposive sampling was conducted with the aim to create maximum-variation representation within the group, e.g. to include participants with different socio-geographical-cultural backgrounds respecting gender, age, religion, marital status, and place of upbringing. A key-informant, the head of department at each college facilitated identification of eligible students, who were invited to participate. All included participants were between 22 and 25 years old. Thirteen respondents were men and ten were women, none were currently in a relationship. Most confessed to Hinduism, three were Buddhists, one Christian and one Muslim. A few had been sexually active. Oral and written information was given about the study, and participation was voluntary and anonymous. All interviews were conducted in privacy.

### Data collection

A topic guide with open-ended questions and probes was developed to explore students’ attitudes and perceptions toward abortion and provision thereof, medical abortion, and task shifting in comprehensive abortion care. The interview guide was subject to constant revision as information from one interviewee influenced the interviewer’s knowledge of the subject [35, 36].

The first author, a Swedish Obstetrician-Gynaecologist who had been residing in India for two and a half years at the time of the study, conducted the interviews in English, the language of tuition at the included colleges. The interviews were conducted in three batches with two larger cities covered during December 2012, one city visited February 2013 and two smaller cities and a peri-urban area in April 2013. The interviews were tape-recorded and lasted between 30 min to one hour. Field notes were taken. Between interviews initial analysis and development of interview guides and probes was conducted. Interviews were continued until theoretical saturation was reached.

### Data analysis

The data was analysed using an inductive thematic approach applying a realist framework to report the experience, meaning and reality of the participants [37]. The recorded data was transcribed verbatim, the transcripts were read through and the recordings repeatedly listened to by the first author. Data was coded manually and organized into units of analysis with a semantic, explicit, approach to identify patterns and themes. The first author conducted the coding and initial analysis; subsequently data and results were reviewed and evaluated by all authors.

### Ethical considerations

The study was conducted in accordance with the World Medical Association Declaration of Helsinki [38]. Administrative permission to conduct the study and disseminate the results was granted from each of the sampled colleges. Informed consent in writing was obtained by all interviewees. Ethical approval was also obtained from the Regional Ethical Review Board in Stockholm, Sweden 2013/415-31/4.

## Results

**Fig. 1:**
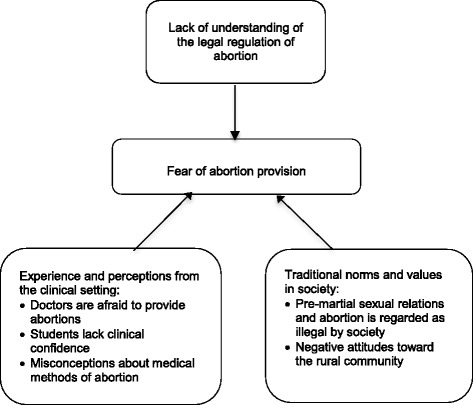
Influences on Maharastra medical students’ fears to provide abortion

The main finding of our study is that the interviewed medical students in Maharastra are afraid to provide abortions in their future practice. This finding is supported by three sub-themes: lack of understanding of the legal regulation of abortion, experience and perceptions from the clinical setting, and the influence of traditional norms and values. Figure 1 illustrates the main theme and sub-themes. A separate theme was generated containing the students’ views on expanding the provider base to non-physician caregivers. The themes are described in detail below.

### Fear of abortion provision

#### Lack of understanding of the legal regulation of abortion

Although interviewees demonstrated knowledge of the MTP act governing abortion, most concluded that abortion is illegal in the state of Maharastra. All respondents discussed the declining sex ratios in India and Maharastra, and the concept of induced termination of pregnancy was commonly intertwined with sex-selective abortion. The legal frameworks regulating those practices were not differentiated. Recent government actions to limit sex selective abortion and especially measures taken against physicians providing such services were well known, and a fear among doctors to be falsely prosecuted for illegal activity was frequently described. The legal conditions for providing medical termination of pregnancy were often correctly cited, but the students also included prerequisites perceived necessary to protect the provider such as obtaining consent from the woman’s husband or parents, even when she was not a minor. One interviewee described “there is fear of abortion both in society and in the mind of doctors” to explain how the on-going campaigns to limit sex-selection influences both people and physicians. Respondents stated that they were not willing to risk their license to practice medicine by providing abortions.

Abortion is banned in India nowadays. It is actually a crime. Since 2 years back, a drive is going on and abortion is a crime in India. Doctors are being jailed for doing abortions illegally. [Informant number 20, Private College, man,]

I understand what her problems are. There are social problems, medical problems and legal problems. In a rape case, or if it is contraceptive failure, then I can manage it.... I do not want to do abortions, I would rather refer to a higher hospital, because I do not want to take the risk to be suspected of illegal actions. Then the government could take my licence. This is why no doctor performs abortions, neither at private hospitals nor at village level. They are referring the patients at higher centres. If the woman has a particular reason for wanting an abortion, then it can be done at a higher centre. [Informant number 12, Government College, man]

### Experience and perceptions from the clinical setting

#### Doctors are afraid to provide abortions

The respondents’ described how their experiences from the clinical setting formed a perception that abortion is illegal. They narrated that doctors were afraid to be prosecuted for abortion activities, and held a fear of adverse events, particularly in primary health centres, and when the patient was an unmarried woman. A general reluctance to provide contraception and abortions among physicians was described, and especially when the patient was an unmarried woman. Respect for officeholders such as police and government officials influenced clinicians’ judgment and decision-making. Accounts of how pregnant, unmarried, care-seekers were reported to the police before being eligible for treatment were common. Some respondents problematized the fear and concluded physicians should be better protected. Physicians in rural areas were often described as lazy, and were believed as not treating patients with attention and respect. Some students had experienced how medical officers at rural health centres rather referred patients to higher centres than provide treatment.

I remember one incident. A 21 year-old girl engineering-student came to this clinic alone with three weeks amenorrhea [since expected first day of menstruation]. She wanted to abort the child... It was such a confidential matter that the senior physician could not handle it. I think she was told to inform the police first. Then I don’t remember what happened. [Informant number 13,Government College, Man]

#### Students lack clinical confidence

Hesitance to provide abortion was enhanced by the student’s lack of confidence in their rapport with the patients. Many participants worried about not obtaining womens’ correct medical history, thus making incorrect decisions regarding treatments and ultimately leading to reprisal. Male students were more uncomfor in rapport to female patients, a male student described, “women are coming to doctors and telling their problem, but I feel uncomfortable about it”. Male students also worried about the patient’s relatives’ reactions if a pelvic exam should be undertaken. Observing physicians’ dependability supported this lack of clinical confidence and fear about taking their own decisions.

My knowledge is good, but my interaction with the patient is not. When I ask them about their problems, they are not ready to tell me. How could I make them talk with me? Even when I ask the questions in the layman language, which they understand, they don’t answer. And if they answer, they don’t tell the truth. [Informant number 22, Government College, woman]

#### Misconceptions about medical abortion

Knowledge gaps and misconceptions about medical methods of abortion were common, medical abortion was confused with emergency contraception and many students’ believed mifepristone and misoprostol are illegal in Maharastra. Women were considered ignorant and not trusted to make their own choices about methods of abortion. It was perceived that most women are afraid of surgery and thus prefer medical methods of abortion. One female student described this by saying “I think women prefer medical abortion, because nobody likes anything being thrust there”. Despite the perception that women prefer medical abortion, dilatation and curettage was seen as the method of choice because respondents feared complications including incomplete abortion and bleeding and doubted women would come to follow-up visits.

Women do not know what is best for them. We can change their attitudes and give them knowledge about what is best for them. For instance, if they come for within 63 days of pregnancy, we can tell them that they can use the medical method, but we can also tell them the disadvantages such as if they take the medication and the pregnancy continues, there would be abnormalities in the baby. [Informant number 14, Government College, woman]

Dilatation and curettage is a good method, sometimes the women take the abortion pill and they don’t come for follow up. Sometimes the products of conception get retained in the uterus. Women have bleedings and other complications but they don’t come back. So if you give them medical termination of the pregnancy they should come for follow-up and ultrasound. They should get information that the uterus is completely empty. But they don’t come back. So therefore dilatation and curettage is better. But gentle curettage..*.* [Informant number 22, Government College, woman]

### Traditional norms and values in society

#### Pre-martial sex and abortion is regarded as illegal by society

Students stated that pre-marital sex and abortion is illegal in society implying that such relations and actions are illegitimate. Those traditional norms and values also affected womens’ access to contraception. Students expressed views such as unmarried women don’t need contraception, that they were dependent on their partner to obtain contraception, and should they use any contraception they were limited to barrier methods. Societal values were given equal importance to the physicians’ decision-making as the legal regulation, especially when unmarried women were concerned. Interviewed female students described how their choice of partners and whether to have sexual interactions were affected by such norms and traditions, and a female student explained that her marrying an inappropriate partner could socially harm her parents. The practice of sex selective abortion was condemned by most, but some male students found such abortions justified in families already having a daughter.

People depend on our culture, it is our roots and our traditions. Your family is the most important and traditional values are honoured. You are not supposed to marry somebody against your cast or religion, it is a very big thing... My parents tell me that I can marry anybody, but they worry that if I did, people would talk behind our backs. It is not their fault, they just want me to be happy. [Informant number 17, Private college, woman]

You cannot have sex before marriage in India, it is not legal. The government can object, and family members tend to hide it to protect their honour and all. [Informant number 23, Government College, man]

#### Negative attitudes toward the rural community

The respondents perceived a social distance from the rural people, whom they believed lacked knowledge and held poor attitudes. They admitted to holding a fear of being threatened and punished by family members, relatives, or fellow villagers should they examine or treat a woman seeking care alone was common, even among students claiming a rural background. Some described how their attitude towards rural people had changed during rotations in rural health centres. Based on their attitudes toward rural people several interviewees stated they would not provide abortion care services in the rural areas.

After my first and second year [in medical school], I believed I should do something for my village. They are poor people. I could change their attitude and teach them. But now, in my final year and internship... I have seen the attitudes of the people who are living in the villages, they are not going to change. They would harass you and could even kill you to maintain their attitudes. I have seen such cases. That is why I don’t want to work in a village. [Informant number 12, Government College, man]

#### Attitudes toward task shifting in comprehensive abortion care

We explored the students’ attitudes toward task shifting in comprehensive abortion care. All participants’ recognized the need to increase the numbers of certified and trained abortion providers in order to improve access to safe abortion in India. Medical staff such as nurses were acknowledged to have substantial knowledge as they had been observed providing services in the field of reproductive health care, such as insertion of intra-uterine devices (IUDs) and counselling. The local connection of many nurses was also seen as an advantage of task shifting. Based on such observations many students recognized that nurses have a potential as abortion providers, presuming they have the appropriate training. A small number of interviewees were reluctant to such task-shifting and found that physicians only should provide such services in the future, as they assumed non-physician providers lack knowledge and an acclaimed need to keep medical expertise and authority by physicians.

So I would definitely support the idea of nurses taking up the physicians’ duties [like abortion provision], creating awareness regarding the population explosion and methods of contraception. I would support task shifting. [Informant number 8, Government College, man]

If nurses are trained properly then they can provide abortion.

[Informant number 21, Government College, man]

Nurses are able to provide abortion, but it shouldn’t be legalized... They can technically provide abortions, but it can result in the misuse of that authority. Providing abortion should be limited to doctors, because they have the right knowledge. It is more appropriate as they can treat adverse events such as infections due to abortions, which nurses wouldn’t understand. [Informant number 22, Government College, woman]

## Discussion

Our study reveals that medical students in Maharastra are afraid to provide abortion services in their future practice. The respondents falsely believed that abortion is illegal, lacked the knowledge to make independent clinical decisions and felt the need to adjust future clinical practice to avoid blame and reprisal. Although the interviewed students were aware of the need to reduce numbers of unsafe abortions in India, and expressed a wish to educate the people in family planning and contraception, they did not recognize their own potential as future abortion providers, and did not acknowledge that their reluctance to provide safe abortion creates a barrier to women’s access to safe services.

The interviewed students were well read regarding details in the MTP act but did not differentiate towards the PC-PNDT act regulating the use of diagnostic techniques to determine foetal sex. It has previously been shown that following the implementation of the PC-PNDT Act, and the enforced implementation thereof, physicians are increasingly unwilling to provide abortions. Our study reveals that this hesitance affects medical students as they depend on their supervising physician to interpret the context and correctly apply the law to the clinical reality.

Medical methods of abortion have been shown to be feasible in the Indian setting and suggested as a means to increase access to safe abortion in India, yet providers lack knowledge and implementation has been slow [39]. Our respondents lacked experience of medical methods of abortion and claimed that dilatation and curettage was the preferred method. which contradicts WHO recommendations. This perception was based on the respondents’ experiences from clinical practice. Studies carried out in India have shown that knowledge and practice of medical methods of abortion is limited among registered providers, yet women have been shown to be positive to medical methods of abortion [40]. A recent study from Madhya Pradesh showed that medical abortion pills were available to women without prescription in over 70 per-cent of surveyed pharmacies [41]. Increased knowledge and training in medical methods of abortion has been show to increase provision at registered facilities, thus improving access to safe abortion.

Tradition and norm are of great importance in the Indian society and lack of interaction with the rural population creates social distances and ultimately fear of physical assault that hinder willingness to perform services. A study from Andra Pradesh and Uttarkhand showed that medical students were concerned with contextual factors, including safety and ability to blend with the rural community, when considering work in rural areas. In-service physicians in rural areas were less affected by contextual factors, which confirms the importance of practical in-service training [42].

Despite several attempts to reform the Indian medical curricula, education remains theory-driven, and lack of practical training is common [43]. Experience and training are determinants of willingness to perform abortion and training in abortion procedure is positively correlated with increased confidence [44, 45]. Studies from different settings, including one from Malaysia, have shown that a majority of medical students are positive to increased training in abortion during medical education [46]. Senior physicians are important role models in the clinical part of the medical education and their practices influence future physician actions and choices. Observing physicians’ insecurity and dependability increased the student’s lack of clinical confidence as well as ability to apply their knowledge and make use of their own judgment. It is important to acknowledge how malpractices and neglect to perform duties influence medical students’ views and future practice. Increased practical training and a more permitting attitude in medical education could reduce fear as a barrier to abortion provision, as well as impact students’ judgment and independent decision-making.

Nurses were acknowledged for their skills, and students were on the whole positive to include non-physician providers in comprehensive abortion care. Increasing the provider base for abortion provision to mid-level providers has been shown to be feasible in the Indian context. A recently published study from Maharastra and Bihar found that allopathic physicians were less supportive than other potential provider cadres to task shifting of medical abortion [47, 48].

The rich findings from these in-depth interviews are a unique complement to our previous study that confirms and further explains the negative attitudes to abortion held by medical students in Maharastra. Lack of knowledge and training creates attitudes and perceptions leading to a fear of abortion provision, thus imposing a barrier on womens’ access to safe abortion services that could easily be avoided.

### Methodological considerations

Trustworthiness was sought throughout the research process. All interviews were conducted in English by the first author who is familiar with the Indian culture and local English dialects. Credibility was strengthened by the use of quotes. Confirmability was assured by summarizing and confirming the meaning of what was said with the respondent, this approach was chosen since the students were in transition to other postings and would be unreachable for member check. Construct and content validity was assured through the main researchers pre-understanding of the context and use of field notes to add richness to data and constructs, as well as the emerging study design with constant revision of the study instrument. Although heterogeneous sampling was conducted the results of our study only reflect the present context. Considering the diversity of Indian states, it is difficult to draw conclusions about the transferability of results, even within the country.

## Conclusion

Interviewed medical students believed abortion is illegal in Maharastra and were afraid to provide abortion care services, as they feared reprisal. It is necessary to strengthen future physicians’ confidence and knowledge through improved medical education in abortion services. Knowledge of legal framework and value clarification related to abortion and clinical skills would increase the probability that medical students include safe abortion services in their future practices.
